# Bioactive Compounds and Quality of Extra Virgin Olive Oil

**DOI:** 10.3390/foods9081014

**Published:** 2020-07-28

**Authors:** Cecilia Jimenez-Lopez, Maria Carpena, Catarina Lourenço-Lopes, Maria Gallardo-Gomez, Jose M. Lorenzo, Francisco J. Barba, Miguel A. Prieto, Jesus Simal-Gandara

**Affiliations:** 1 Nutrition and Bromatology Group, Department of Analytical and Food Chemistry, Faculty of Food Science and Technology, University of Vigo, Ourense Campus, E32004 Ourense, Spain; cecilia.jimenez.lopez@uvigo.es (C.J.-L.); maria.carpena.rodriguez@uvigo.es (M.C.); c.lopes@uvigo.es (C.L.-L.); 2 Centro de Investigação de Montanha (CIMO), Instituto Politécnico de Bragança, Campus de Santa Apolonia, 5300-253 Bragança, Portugal; 3 CINBIO, Universidade de Vigo, Department of Biochemistry, Genetics and Immunology, Campus Universitario Lagoas Marcosende, 36310 Vigo, Spain; mgallardo@uvigo.es; 4 Meat Technology Centre Foundation, 32900 San Cibrao das Viñas, Spain; jmlorenzo@ceteca.net; 5 Nutrition and Food Science Area, Preventive Medicine and Public Health, Food Science, Toxicology and Forensic Medicine Department, Universitat de València, Faculty of Pharmacy, Avda, Vicent Andrés Estellés, s/n, Burjassot, 46100 València, Spain; francisco.barba@uv.es

**Keywords:** extra virgin olive oil, chemical composition, bioactive substances, EVOO quality, applications

## Abstract

(1) Background: Extra virgin olive oil (EVOO) is responsible for a large part of many health benefits associated to Mediterranean diet as it is a fundamental ingredient of this diet. The peculiarities of this golden, highly valued product are in part due to the requirements that must be met to achieve this title, namely, it has to be obtained using exclusively mechanical procedures, its free acidity cannot be greater than 0.8%, it must not show sensory defects, and it has to possess a fruity taste. (2) Methods: All these characteristics are key factors to EVOO quality, thus the chemical composition of these many health-promoting compounds, such as unsaturated fatty acids (which are also the major compounds, especially oleic acid), as well as minor components such as tocopherols or phenolic compounds (which behave as natural antioxidants) must be preserved. (3) Results: Due to the presence of all these compounds, the daily consumption of EVOO entails health benefits such as cardioprotective, antioxidant, anti-inflammatory, anti-tumor properties or acting as regulator of the intestinal microbiota, among others. (4) Conclusions: Taking all together, conserving EVOO chemical composition is essential to preserve its properties, so it is worth to control certain factors during storage like exposure to light, temperature, oxygen presence or the chosen packaging material, to maintain its quality and extend its shelf-life until its consumption.

## 1. Introduction

*Olea europaea* L., commonly known as the olive tree, is a small tree species typically distributed by the Mediterranean countries [1]. Olive oil, its main derived product, has experienced an increase in its popularity due to its organoleptic characteristics and its associated beneficial health effects [2]. The olive tree is one of the species that was first cultivated. In fact, there are records for the first cultivated trees which date from 6000 years in Asia Minor regions according to the International Olive Council (IOC) [3]. Its origin is linked to Mediterranean civilizations and regions, characterized by soft and rainy winters and hot dry summers [4]. Although its industry uses large areas for the cultivation of trees, nowadays, its cultivation has been spread to regions of all continents (excluding Antarctica). Still, Mediterranean countries remain the main olive producers, led by Spain, Italy, and Greece. In consequence, the European Union (EU) is responsible for the 70% of the global olive production, generating a production value of 7000 million euros each year, becoming a key factor for the agro-industrial sector development and thus, a social and economic engine for the EU southern regions [5].

The French writer Georges Duhamel once wrote “There, where the olive tree gives up, is where the Mediterranean ends. The tree of light is the nature and culture of the Mediterranean” [6]. This statement corroborates the joining and simultaneous development of a culture-bound to olive products and particularly olive oil. So much, that olive oil is a crucial part of the commonly known as Mediterranean diet (MED). This diet consists of a balanced combination of low animal protein consumption with a high intake of fruits, vegetables and cereals and olive oil as the principal source of fat in many foods [3]. MED has been usually associated with a low incidence of cardiovascular diseases (CVD) risk in EU southern regions, which likewise, show higher life expectancy when compared to EU northern countries and the United States of America (USA) [7,8]. Several studies have been conducted to evaluate and prove MED as the main protective agent in the primary prevention of chronic diseases. Research has pointed out that the beneficial health effects of MED are attributed to a large extent to olive oil.

**Figure 1:**
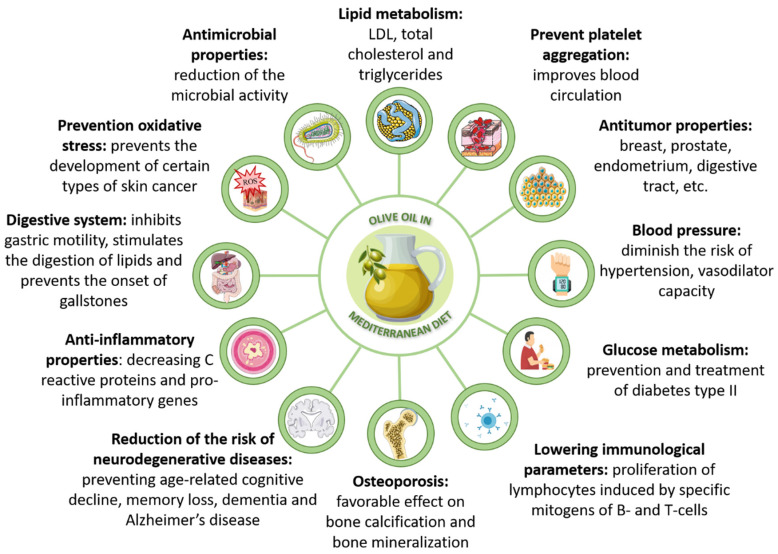
Scheme of the beneficial and healthy effects of the olive oil inclusion in the Mediterranean diet. Information adapted from [11,12].

Some of those beneficial properties are gathered up in Figure 1. Its unique composition and biological properties are largely responsible for this association. Olive oil composition is mainly formed by triglycerides and a variety of several compounds in small quantities. Among the glyceride fraction, olive oil shows a high content of fatty acids and particularly, an elevated proportion of monounsaturated fatty acids (MUFA). Unsaturated acids are up to 85% of its composition, due to its high content in oleic acid (C18:1), which might range between 70–85% and other fatty acids as linoleic or palmitoleic acid. In the case of saturated fats, they entail around de 14% of oil composition, basically due to palmitic and stearic acids. Regarding minor compounds, they signify less than 2% of olive oil composition, and the best representatives of this group are phenolic compounds, although this minor group also includes some lipophilic compounds like α-tocopherol (vitamin E). Likewise, there are several hydrophilic phenolic compounds, among which the simple phenol hydroxytyrosol and the secoiridoid oleuropein must be highlighted [1,8]. Besides, olive oil is also a source of pigments like carotenoids [3]. Phenolic compounds are known for their biological properties. In particular, these olive oil compounds have shown potential as antioxidant, anti-inflammatory and antimicrobial agents [9]. However, their concentration is related to different factors: olive varieties, environmental factors, time of harvest and extraction, and storage conditions [1]. To sum up, olive oil is considered as a superfood due to its health properties derived from its unique composition; this is, its lipid profile and its bioactive compounds content [10]. Moreover, its singular composition has attracted so much attention that olive oil has been studied as a source of these bioactive compounds but also their derived residues have shown high concentrations of these molecules and thus a great potential for industrial recovery and related applications, such as formulation of new high added value products [10].

**Figure 2:**
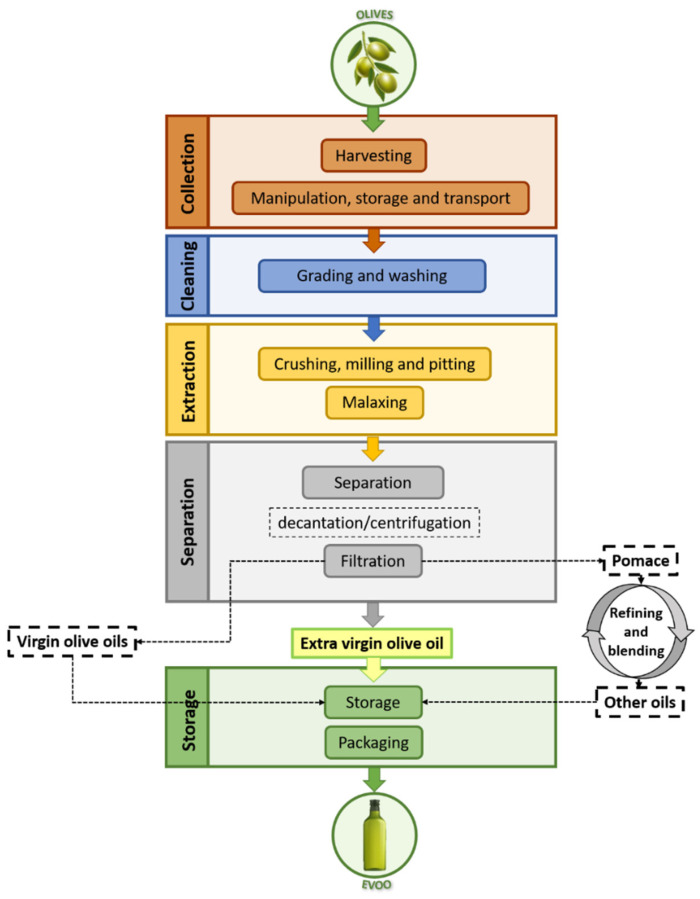
Summary of the oil extraction process focused on extra virgin olive oil (EVOO) production. The boxes with discontinuous outline refer to the obtainment of other type of oils. Modified from different schemes of [10,17].

Figure 2 shows a detailed procedure of the extraction of olive oil from *Olea europaea* L. of different varieties, whose extracting process follows a common line formed by different steps: collection, grading, washing, crushing, malaxing, separation and centrifugation, storage and packaging. This process is not aimed at improving oil quality; however, attention must be paid to its development to not cause a loss of quality [13,14]. Depending on the modifications of this process and the resultant organoleptic characteristics of the product, among others, the EU defines on the Article 118 of European Commission (EC) Regulation (EC) No 1234/2007 six main types of oils, which are delineated also by the IOC [15]. The specific attributes of each type of oil are shown in Table 1. Regarding all these groups, virgin olive oil (VOO) must be highlighted due to their organoleptic characteristics and beneficial properties. Among VOOs, the EU establishes three types of oils: extra virgin olive oil (EVOO), VOO, and lampante olive oil. Furthermore, IOC adds an extra subgroup called ordinary virgin oil. All VOOs are characterized for being obtained by mechanical processes (only washing, decantation, centrifugation and filtration) under specific thermal conditions which do not cause any alteration. Afterward, they are divided according to their acidity, which gives an idea of the content in free fatty acids based on the percentage of oleic acid. Lower acidity values guarantee a high-quality oil, showing it has been obtained from healthy olives and under ideal conditions. Moreover, they are submitted to sensory analysis to asses some requirements [16].

Particularly, EVOO is obtained directly from olives, that is, pure olive juice. It is considered the highest quality oil, and in general, it is characterized for having a low acidity, up to 0.8% and a sensory grade higher than 6.5 points, thus, having perfect aroma and flavor [3,10]. Regarding sensory aspects, EVOO must show a fruity note higher than 0 and, more importantly, a median of zero defects [18]. In addition, and to offer the most possible information to the consumer, these oils are usually labeled as intense, medium or light depending on their positive attributes [19]. However, sensory analysis is always more difficult to unify. On the other hand, EVOO is susceptible to different chemical alterations during its production. Hydrolytic and oxidative degradation are the main causes of undesirable product formation so that water plays an important role since lipases (naturally present at olive pulp or seeds) are active in an aqueous phase. Lipolytic reactions lead to the formation of free fatty acids, thus increasing the acidity of the oil. To avoid these reactions, the filtration step is of high importance, as well as paying attention to other factors such as avoiding microorganisms or mechanical crushing, among others. Moreover, water may affect the transformations of different phenolic compounds [18,20]. Regarding oxygen, EVOO must have a peroxide index (mEqO_2_/kg) ≤20. Oxygen is crucial for the lipoxygenase cascade reactions and its consequential positive sensory notes, but its excess can cause defects [20]. Moreover, EVOO must also show specific spectrophotometric values: absorption λ = 233 nm, ≤ 2.50 and λ = 270 nm, ≤ 0.22, which are quality and authenticity indicators, respectively [18]. Besides, contaminants (i.e., mycotoxins or pesticides, among others) are another crucial factor in avoiding associated risks [21]. Considering all the characteristics that EVOO is required to fulfill, quality control is a must. Both IOC and EC have approved different standards in matters of purity (oil composition) and quality (organoleptic profile, acidity, peroxide value, among others) as well as sensory analysis to ensure that alterations have not occurred during the process [10]. Additionally, another remarkable aspect is traceability. Traceability must be assured during all the obtaining process of EVOO, including the four basic steps (harvesting, milling, storage, and packaging batches) of the procedure to guarantee the identity of the product and its production chain. This approach also leads to the next step, this is certification, carried out by normalization and certification quality organisms in order to prove the rigorous process system employed at EVOO production [22,23].

Regarding its composition, EVOO is mainly composed of triglycerides (97–99%) and minor compounds (1–3%), which are the principal responsible for its biological properties and sensory attributes. It has a high content of MUFA (65–83%), especially oleic acid, and some polyunsaturated fatty acids (PUFA) such as linoleic acid, which is considered a potent fatty acid on the reduction of low-density lipoprotein (LDL) cholesterol. This lipid profile and also high ω6/ω3 ratio have been linked to protective effects on coronary, autoimmune and inflammatory disorders but also as anti-thrombotic and regulators of blood pressure [24,25,26]. Concerning bioactive compounds, their main representatives are the same of oil in general, namely phenolic compounds such as hydroxytyrosol and derivatives (oleuropein and tyrosol), tocopherols but also other compounds as hydrocarbons (i.e., squalene) or pigments like provitamin A compounds [3,26]. However, it must be mentioned that some of these compounds such as squalene might be lost during refinery, so they can only be found on this type of oil [25].

As mentioned, all these bioactive compounds are known for their biological properties and positive effects on human health. EVOO inclusion in the diet and its bioactive molecules have been studied to identify its effects. EVOO is known for having a high content of antioxidant compounds with protective properties against free radicals. Therefore, it has been pointed out that its high consumption is related to a generally low risk of suffering colon, breast or skin cancer as well as beneficial effects on aging and coronary diseases [1]. It has also been proposed as a preventing tool of Alzheimer’s and other neurodegenerative diseases [3], as anti-inflammatory [7] and also as immune-stimulating [24]. Another study proved that rats fed with EVOO in substitution of lipids and complemented with physical exercise, could avert cartilage diseases as osteoarthritis [9]. Additionally, EVOO consumption has shown positive effects on gut microbiota [27]. Some studies have researched the bioavailability of phenolic compounds of EVOO and found that 55–60% of them can be absorbed, most of them at small intestine [11]. Moreover, their different compounds have shown other beneficial properties like antimicrobial, antitumor or protective agents against cellular damage [26,28]. More specific studies have also related EVOO treatment with positive gene regulation and with micro ribonucleic acid (miRNA) modulation of target genes associated with synaptic plasticity as well as to motor and cognitive behavior [29]. There are multiple bibliographic references directed towards proving all these promoting effects. Nevertheless, a specific study must be highlighted, the PREDIMED trial (prevention through MED, in Spanish). This is one of the largest nutritional studies ever conducted in Spain, which evaluated the effects on primary prevention of CVD when following a MED supplemented by EVOO or nuts mix [30]. This project groups together different studies, which have brought to light several positive consequences: reduction of CVD risk, reduction of C-reactive protein, reduction of atrial fibrillation, prevention of diabetes and metabolic syndrome, reduction of diastolic blood pressure, higher protection against breast cancer or lower prevalence of non-alcoholic fatty acid liver disease [25,31,32,33]. However, more epidemiologic studies and controlled trials are necessary to better validate and understand the beneficial effects of EVOO consumption. At last, it is worth mentioning that new disciplines (encompassed as nutrigenomics) are also working on new approaches for evaluating the health-promoting effects, characterizing new markers, and understanding their action mechanisms [29,34].

Regarding all the presented information, this article aims to review the current knowledge about the unique EVOO composition and its related bioactivities. Therefore, a revision focused on its beneficial and health-promoting properties, its chemical composition as well as some quality parameters is presented. To achieve this purpose, a systematic search in several databases was carried out based on the topic described, all in all, ~5000 documents have been published. Most of the publications correspond to research articles and book chapters, 56.3% and 15.6%, respectively, followed by reviews (12.5%). Among them, bibliographic references were chosen according to their concordance with the covered topics. The extensive information compiled was further classified into the different sections presented in this review. After analyzing the considerable amount of data available on this field, it can be said that EVOO is a current topic; although it has been known and used for centuries, it is still under study, which means EVOO has potential properties and/or applications yet to be discovered.

## 2. Main Components of EVOO

Virgin olive oils are oils obtained from the fruit of the olive tree (*Olea europaea* L.) solely by mechanical or other physical means under conditions, particularly thermal conditions, that do not lead to alterations in the oil, and which have not undergone any treatment other than washing, decantation, centrifugation and filtration [35]. The use of said physical techniques allows the preservation of many compounds that make EVOO one of a kind among plant oils. Its uniqueness is due to the abundance of fatty acids, PUFA and MUFA but also the occurrence of many bioactive molecules, like hydrophilic phenols, phytosterols, tocopherols and carotenes that provide several functional properties as well as a long storage time due to their high oxidative stability [36,37,38]. Other vegetable oils, like palm oil, are rich in saturated fats, which are more stable during the cooking or frying processes than the unsaturated ones, avoiding degradation to toxic compounds, but they do not have beneficial properties for the human health as the unsaturated one. On the other hand, sunflower oil is rich in unsaturated fats, especially in linoleic and oleic acids that enhance its healthy properties but decrease its thermal stability [39]. EVOO has a good PUFA:MUFA balance, which confers it stability properties against oxidative thermal degradation, particularly regarding the formation of volatile aldehydes, so EVOO is a proper and recommendable oil to use in food frying [40]. This relation between PUFA and MUFA and the low content of saturated fats also makes EVOO one of the healthiest vegetable oils to be consumed raw because it helps reduce LDL cholesterol levels in the human body [41].

**Figure 3:**
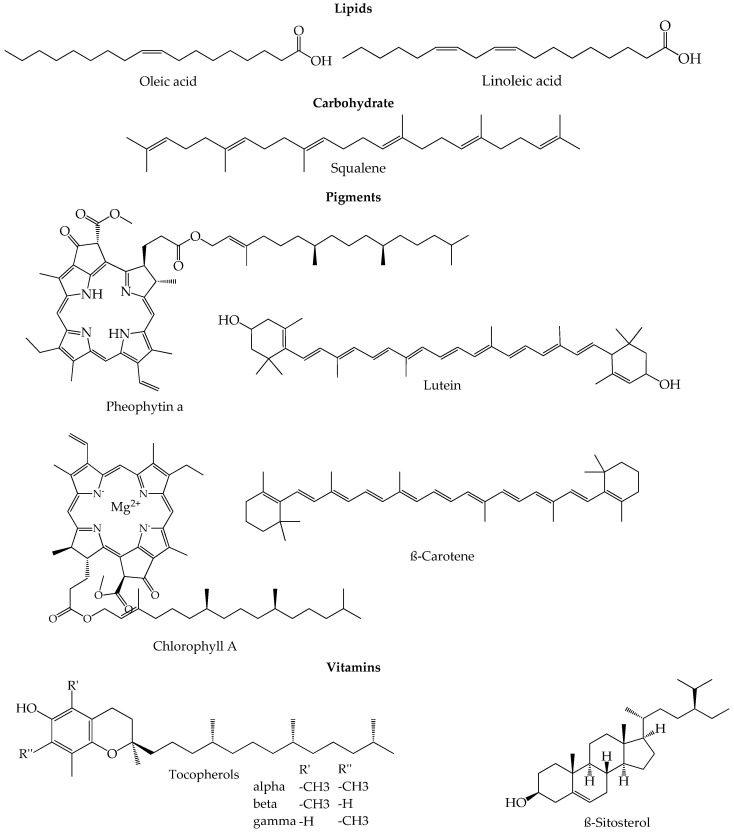
Representative chemical structures of some relevant compounds present in EVOO.

The composition of EVOO is a result of several factors like genotypic potential, environmental factors, fruit ripening, harvest time, agricultural factors (irrigation, sunlight, orchard management) and also technological factors like the method applied for oil extraction or the storage conditions [42]. The concentration of the minor and major fruit components changes and depends on all those variables. Apart from that, the olives ripening process lasts a few months in which the atmospheric, environmental and agricultural conditions play a very important role despite the strict genetic control that can be applied [43,44]. During the maturation and ripening process, the photosynthetic activity decreases as the oil content in the fruits increases [45]. In the first stage of ripening, denominated green stage, the ripe fruits have already acquired their final size, so the maturation proceeds, and the chlorophylls in the skin are slowly swapped by anthocyanins, turning the olives from green to dark violet or purple until the end of the ripping process. These changes in color define the spotted, purple and black stages [43,46]. Olives have the highest phenolic compound content at the phase between green and darker skin, and therefore, the degree of maturation is an important factor to define the right harvest time that will originate the best quality olive oil [47]. Figure 3 shows a summary of representative chemical structures of some relevant compounds present in EVOO.

### 2.1. Primary Metabolites

#### 2.1.1. Lipids

Lipids are one of the principal sources of energy for all living beings and additionally, they are also involved in many physiological functions, as their role as a structural component of cell membranes, nervous system, the production of hormones, brain development and also on the promotion of liposoluble vitamins absorption.

EVOO is widely used in the human diet, especially in the MED and has been long renowned for its many health-promoting properties. Its consumption is associated with reduced risk of several chronic illnesses, like diabetes, hypertension, obesity and CVD [48,49]. These health properties are related to the presence of bioactive compounds like phenolic compounds but also with the high content in MUFA. Olive oil has a high content in oleic acid transforming it into a healthy fat, especially when compared with other vegetable oils [40]. This lipid can decrease the risk of CVD due to its effects on the lipids present in the blood vessels [50]. According to available data, there is 65.2–80.8% of MUFA in the lipidic fraction of olive oil [51]. Other fatty acids found in the total fatty acids composition of olive oils are palmitic acid, palmitoleic acid, stearic acid, linoleic acid, α-linolenic acid, and other minor ones that are listed in Table 2.

Triacylglycerols constitute a big part of the edible oil and a high percentage of the saponifiable fraction is constituted by MUFA [27]. The principal triacylglycerol detected in olive oil is oleic–oleic–oleic (OOO), representing about half of the total triacylglycerol portion found in EVOO. Other triacyclglycerols also present are palmitic–oleic–oleic (POO), oleic–oleic–linoleic (OOL), palmitic–oleic–linoleic (POL) and stearic–oleic–oleic (SOO) [43,52]. Diacylglycerols and monoacylglycerols have been identified in VOO at concentrations of 1–2.8% and 0.25, respectively [53].

Four classes of sterols also take place in olive oil and are commonly used to check its genuineness because their presence is linked to the quality of the oil. These four classes are common sterols (4-Desmethylsterols), 4α-Methylsterols, triterpene alcohols (4, 4-Dimethylsterols) and triterpene dialcohols [43]. Common sterols in EVOO are present in both free and esterified forms [59]. The leading components of this sterol fraction are campesterol, β-Sitosterol and Δ5-Avenasterol [60,61], and in smaller amounts, it is also possible to find stigmasterol, cholesterol, cholesterol, brassicasterol, sitostanol, ergosterol, campestanol, Δ7-Cholestenol, Δ7-Avenasterol, Δ7-Stigmasterol, Δ7-Campesterol, Δ5,24-Stigmastadienol, Δ5,23-Stigmastadienol, Δ7,24-Ergostadienol, Δ7,22-Ergostadienol, 22,23-Dihydrobrassicasterol and 24-Methylene-cholesterol [62,63]. The total sterol content of EVOO varies between 1000 and 2000 mg/kg, being the first value the inferior limit set by the EU Commission [43]. β-Sitosterol is the main compound in the sterol fraction with values between 75% and 90% of the total sterol fraction, while Δ5-Avenasterol has values between 5% and 20% [62]. Crop year, cultivar, ripeness of the fruit, storage time of the olives before oil extraction and geographic influences all contribute to sterol composition of the final EVOO obtained [64,65,66]. At the same time, storage time and conditions of the final product are also factors that can originate several important changes particularly in the concentrations of each individual sterol [43]. 4-Monomethylsterols are present in smaller amounts and signify part of sterol biosynthesis as intermediates. They can be found in their free and esterified forms [67]. The most common are gramisterol, obtusifoliol, cycloeucalenol and citrostadienol [60,62], and their concentrations vary between 50 and 360 mg/kg of oil [60,68]. Triterpene alcohols, also identified as 4,4-Dimethylsterol, are a very complex fraction that can be in free and esterified form, and whose main compounds are butyrospermol, β-Amyrin, cycloartenol and 24-Methylenecycloartanol. In smaller amounts or trace quantities, cyclosadol, cyclobranol, dammaradienol, germanicol, 24-Tirucalladienol, 24-Methylene-24-Dihydroparkeol, α-Amyrin, taraxerol, 7, parkeol and tirucallol can also be found [62]. Total triterpene alcohol levels range from values of 350 to 1500 mg/kg [68,69]. Lastly, among the triterpene dialcohols class, erythrodiol (5α-olean-12-ene-3β, 28-diol, homo-olestranol) in free and esterified form and uvaol (Δ12-Ursen-3β,28-diol) are the major triterpene dialcohols found in EVOO [70], and their presence is mainly affected by cultivation characteristics [68]. EVOO contains levels of total erythrodiol from 19 to 69 mg/kg of oil, and the free form is inferior to 50 mg/kg [59,68].

#### 2.1.2. Tocopherols

Three isoforms of tocopherols are present in EVOO: α-, β- and γ-tocopherol. α-Tocopherol can be found in its free form and represents more than 90% of the identified section with ranges from 206.5 to 270.9 mg/kg of oil to 191.5 to 292.7 mg/kg of oil, values that fluctuate with variables as the year of harvest and spacing between olive trees [71]. Both, the distance between plants and the crop year influenced statistically tocopherols amount [43,71]. Besides, the high levels of this type of tocopherol may be linked to the high levels of chlorophyll pigments and the simultaneous necessity for singlet oxygen deactivation [72].

Lower quantities of β-Tocopherol (~10 mg/kg), γ-Tocopherol (~20 mg/kg) and δ-Tocopherol (~10 mg/kg) can also be found on EVOO. The total tocopherol concentration seems to decrease in the ripping of the fruits, and the refining or the hydrogenation process causes their degradation, so they are only found in the EVOO and VOO [73].

#### 2.1.3. Carbohydrates

There are two hydrocarbons mainly present in olive oil, 2,6,10,15,19,23-Hexamethyl-2,6,10,14,18,22-Tetracosahexaene also known as squalene and β-Carotene, which will be addressed in the pigments section of this review. Squalene is the last metabolite synthesized before the sterol ring formation. Some of the beneficial health effects of olive oil are partially linked with the presence of squalene, and it has also demonstrated antitumoral effects against certain types of cancer [74,75]. This compound constitutes more than 90% of the hydrocarbon fraction and is the most abundant compound in the unsaponifiable matter, with concentrations ranging from 200 to 7500 mg/kg oil [57]. In a different study, squalene was reported in even higher concentrations, up to 12,000 mg/kg oil. Squalene content depends on several variables like the type of olive cultivation and the oil extraction technique applied, and it decreases largely during the refining process so it is present in larger quantities in EVOO and VOO [76].

The remaining fraction of carbohydrates in EVOO is composed of triterpene and diterpene, isoprenoid polyolefins, hydrocarbons and n-paraffins [43,76].

### 2.2. Secondary Metabolites

#### 2.2.1. Phenolic Compounds

The principal group of antioxidants in EVOO are hydrophilic phenols, and these compounds are extremely relevant when it comes to determining the quality of the oil regarding their sensory characteristics, like bitterness, pungency and stability [38,77], as well as determining the organoleptic characteristics of aroma and flavor of each EVOO [53]. The oxidative stability of EVOO depends not only on the olive variety and quality but also on the harvesting time; cultivation area; the degree of unsaturation and the levels of antioxidants present from tocopherols, hydrophilic phenols and carotenes. Besides, factors like oil extraction system and storage conditions also influence its conservation [78].

The correlation of the phenolic content of olive oil and oxidative stability was studied showing that these two are interconnected [79]. Furthermore, EVOO phenolic compounds provide benefits for human health in the prevention of several chronic diseases [80,81]. Various studies indicate that EVOO phenolic compounds have antioxidant, anti-inflammatory, antimicrobial and antitumoral activities, and they can also modulate gene expression to protect proteins that take part in the cellular mechanisms involved in the inflammation process, the oxidative stress resistance and in lipid metabolism [82,83]. Therefore, the major antioxidant substances found in EVOO are polar phenolic compounds that can be present in free, bound or esterified forms [43], and usually, its total phenolic content ranges between 50 and 1000 mg/kg [84], being more common in concentrations between 100 and 300 mg/kg [43]. Likewise, each EVOO has a different phenolic profile, content and composition due to the differences discussed above [78].

Phenolic compounds have been largely reported in EVOO composition, with more than 30 different compounds identified [47,85], being the major phenolic acids present in EVOO hydroxybenzoic, *p*-Coumaric, ferulic, gallic, syringic, vanillic, caffeic, *o*-coumaric and sinapic acids [53]. Other types of polyphenols that can also be found in EVOO are flavonoids, lignans, hydroxy-isocromans, secoiridoids and phenolic alcohols. The major flavonoids found in EVOO are luteolin, apigenin and many of their derivates [86,87], whereas the main lignans present are (+)-pinoresinol and (+)-1-Acetoxypinoresinol [88], being the usual lignan content in EVOO between 1 and 100 mg/kg [89].

Secoiridoids are rare phenolic compounds present in plant species, nevertheless, they are found in abundance in Oleaceae species, particularly in *O. europaea* leaves and fruits. However, they are insoluble in oil and therefore only a small percentage of these compounds ends up in the final EVOO after the mechanic extraction process. Nevertheless, they are one of the most important micronutrients on EVOO for their sensorial and heath properties [38,80]. The most common secoiridoids are demethyloleuropein, oleuropein, ligstroside and their aglycones, the last ones accounting for approximately 90% of the phenolic compounds in EVOO [90]. Secoiridoids are hydrolyzed through crushing and malaxation by enzymatic reactions catalyzed by endogenous b-glucosidases yielding secoiridoid aglycons [91]. The bitterness of olive oil is due to the secoiridoids present, especially the dialdehydic form of oleuropein aglycone [92].

Isochromans are only found at low concentration in EVOO, and the two mainly found are 1-Phenyl-6,7-dihydroxy-isochroman and 1-(3′Methoxy-4′-hydroxy)phenyl-6,7-dihydroxy-isochroman [93]. The concentration of these compounds increases during the extraction process because of the hydrolytic process that originates carbonyl compounds and hydroxytyrosol, which are isocromans derivatives [88]. Finally, the principal phenolic alcohols found in EVOO are tyrosol (p-Hydroxyphenyl ethanol) and hydroxytyrosol (2-[3,4-Dihydroxyphenyl] ethanol). These are present in small concentrations in fresh olive oil but tend to increase along the storage process because of the hydrolysis of olive oil secoiridoids [94].

#### 2.2.2. Pigments

The lipophilic carotenoid and chlorophyll pigments occurring in olive oil are responsible for its characteristic color [95]. The coloration of EVOO is greener in the presence of green olives that have higher chlorophyll content whereas using mature olives with higher carotenoid content we obtain a more yellowish oil, so the final color is a result of the proportions of these pigments [96]. EVOO has a large variety of carotenoids and chlorophylls, from β-Carotene, violaxanthin, neoxanthin, lutein and other xanthophylls to chlorophyll a and b, pheophytin a and b and other minor derivatives [97,98]. These pigments can be found in amounts up to 100 ppm of total carotenoids and major pigments like pheophytin up to 25 ppm, β-carotene up to 15 ppm and lutein up to 10 ppm [96], although these values depend on various factors. The final concentration of each pigment in the final EVOO relies on the physicochemical characteristics of the fruit, the geographic origin, climate and irrigation conditions and the mechanic extraction process used. Storage conditions and final packaging also play a role in pigment concentration and type [96,99,100].

Quality and adulteration of EVOO are sometimes analyzed through the measuring of pigment compounds because they are correlated with EVOO nutritional value, freshness and antioxidant properties [99,101]. In addition, pigments can also be used for the authentication of EVOO, by measuring the chlorophyll and carotenoid pigments of EVOO and comparing them through a quality index, in which the total chlorophylls to total carotenoids ratio must be around 1 and the ratio of minor carotenoids to lutein must be around 0.5, to declare it an authentic olive oil [102]. These parameters are valid for any olive oil regardless of the studied variety. Furthermore, other pigments like violaxanthin, lutein and total pigment content can be useful as a tool to identify a monovarietal EVOO [102]. Chlorophylls, carotenoids and other minor pigments like lutein and violaxanthin can be stable for more than one year in storage regardless of the degree of ripeness and variety of the olives used to produce that oil [103].

The degradation of chlorophylls occurs as a consequence of a pheophytinization reaction that starts from the malaxation step during the extraction of the EVOO and increases throughout storage time [104]. During that process, the chlorophylls naturally present (a and b) are slowly but irreversibly converted into pheophytins a and b, where the central Mg^+2^ ion of the porphyrin ring is exchanged with two hydrogen atoms making the molecules more stable. These eventually turn to pyropheophytins by the removal of the carboxymethyl group, which are the ultimate products of chlorophyll degradation [105].

## 3. Biological Properties of EVOO

The Seven Country Study conducted in the middle of the 20th century first demonstrated the cardioprotective capacities and health benefits of MED [106], olive oil being the hallmark of this dietary pattern. Since then, plenty of observational and epidemiological studies have demonstrated the health-promoting effects of consuming olive oil.

Many health benefits of following a MED enriched in EVOO have been reported by the PREDIMED trial [107], such as protection against CVD [30] or oxidative damage [108,109] and prevention of breast cancer [110] and type 2 diabetes mellitus [111]. Many other randomized controlled trials, prospective study cohorts and meta-analysis, supported by in vitro experiments, indicate that EVOO possesses interesting biological activities and pharmaceutical-nutritional properties (Table 3) that exert a beneficial health impact that stands out over other fats and oils. Nevertheless, many attributes of EVOO are also related to the MED, the context in which its beneficial effects have been mainly evaluated.

### 3.1. Cardioprotective Properties

The Seven Country Study started in the 1950s first demonstrated the cardioprotective abilities of MED [106] and has been supported by numerous further studies based both on MED and olive oil consumption. 

More recently, the cardioprotective benefits of a MED enriched with EVOO have been proven by the PREDIMED study. This multicenter, randomized, controlled trial involved ~ 7500 subjects with potential cardiovascular risk, showing no CVD at enrolment. The PREDIMED trial resulted in a 30% decrease of a major CVD development, such as stroke or myocardial infarction, in comparison to a control group that followed a low-fat diet [30,107]. An observational study based on the PREDIMED cohort indicated that consumptions of 10g EVOO/day are related to CVD risk diminutions up to 10% [112]. A recent systematic review evaluating clinical trials reported that diets enriched with 10–50 mL/day of EVOO (but not diets supplemented with EVOO capsules) significantly decreased diastolic blood pressure by 0.73mm Hg [113].

In another meta-analysis of randomized controlled trials, case-control and prospective cohort studies including ~40,000 cases of stroke and ~100,000 cases of coronary heart disease (CHD), it was reported that for each increase of 25 g of olive oil intake, stroke and CHD risk was reduced by 26% and 4%, respectively. When combined stroke and CHD, olive oil consumption also showed preventing effects, decreasing the risk of a CVD event by approximately 18% [114].

The preventive role of EVOO polyphenols against CVD was also documented in a meta-analysis of controlled trials that evaluated the effect of low versus high polyphenol olive oil on markers of CVD risk. Olive oil consumption ranged from 25–75 mL/day. High polyphenol olive oil significantly reduced the CVD-risk markers malondialdehyde, oxidized LDL, total cholesterol, high-density lipoprotein (HDL) cholesterol and also some inflammatory indicators like C-reactive protein (CRP) or interleukin-6 (IL-6) [115].

### 3.2. Antioxidant Activity

The antioxidant effects of EVOO have been deeply analyzed given the correlation between oxidative stress and CVD or atherosclerosis. Evidence from several meta-analyses and randomized controlled trials, such as the EUROLIVE study [117,119], demonstrated in their analyses the reduction of lipid oxidative damage, the LDL capacity to suffer oxidation and a decrease in oxidized LDL concentration after high-phenolic VOO and EVOO intake, in a dose-dependent way [118,120]. It is also worth noting the health claim allowed by the European Food Safety Authority (EFSA) concerning the protective effects of 5mg/day of olive oil phenolic compounds against LDL oxidation [116]. The PREDIMED cohort was also used to evaluate the antioxidant effects of EVOO. The intervention group with MED enriched with EVOO reported an improvement of HDL atheroprotective functions, oxidative status and composition and also increased resistance to LDL oxidation and low grade of LDL oxidative alterations in comparison to the control low-fat diet [108,109].

Pinoresinol and acetoxypinoresinol, phenolic compounds present in EVOO but not in olive fruits or refined oils, isolated from EVOO or other sources such as sesame seed, have reported in vitro antioxidant capacity [121]. The enzymatic hypoxanthine/xanthine oxidase assay reported a higher antioxidant potential of acetoxypinoresinol, compared to the classic antioxidants, vitamin E and dimethylsulfoxide (IC_50_ of 0.91, 12.4 and 2.30 nM, respectively). Pinoresinol possesses the ability to inhibit LDL oxidation but has shown inconsistent results (IC_50_ ranging from 24.6–558 µM, 2,2-Diphenyl-1-picryl-hydrazyl-hydrate free radical assay (DPPH) colorimetric assay).

### 3.3. Anti-Inflammatory Activity

Recurrent or chronic inflammation is a main etiologic factor of several non-communicable pathologies, whose prevalence is promptly increasing. Thus, the anti-inflammatory effects of EVOO have gained attention and so have been widely evaluated.

A recent meta-analysis of randomized controlled trials evaluated regular olive oil intake effects on inflammation [122]. The authors reported a decrease in the levels of IL-6, tumor necrosis factor-α (TNF-α) and CRP, the three plasmatic inflammatory indicators considered. Such beneficial effects were shown in studies when EVOO was regularly consumed for more than 3 months. The overall health status of participants should also be taken into account, as the strongest positive effects were reported among unhealthy groups (with type 2 diabetes mellitus or at risk of CVD). Another meta-analysis comprising 3106 participants also showed a significant reduction of IL-6 and CRP levels, when olive oil was consumed as a supplementary or natural intake [123]. The adherence to a high-phenol VOO breakfast decreased the postprandial inflammatory response, reducing the levels of plasma lipopolysaccharides in patients with metabolic syndrome [124]. The higher polyphenol content of EVOO may mediate the mentioned favorable effect as it has demonstrated anti-inflammatory effects in vitro [133]. The anti-inflammatory effect of phenolic compounds-enriched EVOO has also been reported in the adipose tissue in mice, with anti-atherosclerotic effects [134].

Due to these mentioned capacities, EVOO has also been proposed as a potential therapeutic product, reducing inflammation in inflammatory bowel diseases, including ulcerative colitis and Crohn’s disease, being both related to chronic inflammation of the intestinal mucosa [135,136]. The benefits of EVOO consumption were evaluated in other autoimmune and chronic inflammatory diseases such as rheumatoid arthritis [137], systemic lupus erythematosus [138] or multiple sclerosis [139,140] with promising results in murine models. Besides, both in vitro and in vivo studies outline that the anti-inflammatory activity of EVOO provides a neuroprotective effects that could prevent cognitive decline and, therefore, the development of Alzheimer’s disease or elderly dementia [3,141].

### 3.4. Antitumoral Activity

Traditionally, a lower incidence of cancers such as breast, colorectal, endometrium and prostate cancer has been observed in Mediterranean countries linked to dietary factors, when compared to the USA or other European countries [142]. The antitumoral and anticancer activities of EVOO, as well as of specific fractions or isolated compounds, have been widely studied and evidenced both in vitro with cell cultures and in vivo with animal models, observational cohort studies and clinical trials [143].

Evidence from 19 case-control observational studies, including in total 13,800 cancer cases and 23,340 controls, suggests that olive oil intake is inversely associated with the risk of having any type of cancer (34% lower likelihood of cancer for high olive oil intake) [125]. More precisely, this meta-analysis associated lower odds for developing breast and digestive cancer with olive oil consumption (log odds ratio of −0.45 and −0.36, respectively).

The strongest beneficial effects of EVOO concerning cancer have been described in breast cancer prevention. A meta-analysis reported a statistically significant inverse association between estrogen receptor-negative postmenopausal breast cancer and the adherence to MED [126]. Breast cancer incidence was also included in the PREDIMED trial, which included ~ 4200 women. Those allocated to the MED enriched with EVOO showed a 62% relatively lower risk of breast cancer, compared to women who followed a low-fat diet [107,110]. To our knowledge, no recent large case-control or prospective cohort studies have been conducted about the relationship between colorectal cancer risk and EVOO consumption. Nevertheless, a meta-analysis has determined that MED consumption is related to a 14% lower risk or developing colorectal cancer [127]. Recently, it has been suggested that the antitumoral activity of EVOO, lowering colorectal tumor incidence in rats, could be mediated by epigenetic mechanisms, such as miRNA and deoxyribonucleic acid (DNA) methylation [144].

In vitro experiments have shown that both the phenolic fraction of EVOO and specific compounds such as hydroxytyrosol, caffeic acid, p-Coumaric acid, 1-acetoxypinoresinol and pinoresinol, among others, have antitumoral activity against breast cancer cell lines [128,129]. Other in vitro studies about the cytotoxic effect of the EVOO lignan pinoresinol have reported variable results, depending on the cancer cell line tested. Pinoresinol shows a cytotoxic effect against breast, lung and prostate cell lines, and it inhibits cell viability of colon cancer cells. A synergic effect with other EVOO phenolic compounds have been reported [121,130].

### 3.5. Positive Modulation of Gut Microbiota

Much of the health benefits of olive oil consumption are attributed to the metabolism of the phenolic compounds carried out by the gut microbiota [145]. It is estimated that 90–95% of total phenolic compounds intake is not absorbed in the small intestine; therefore, they remain in the large intestinal lumen where they are subjected to gut microbiota metabolic activities. As a consequence, polyphenols are converted to low-molecular-weight compounds that are absorbed and responsible for the health benefits derived from polyphenol-rich food, such as EVOO [132,146,147]. 

A recent review and meta-analysis of randomized controlled trials supports the prebiotic action of polyphenols, capable of modulating and improving intestinal microbe populations, which affects to CVD and colorectal cancer markers [132]. Furthermore, another randomized controlled trial showed that the ingestion of VOO enriched with phenolic compounds decreases the serum levels of oxidized LDL in hypercholesterolemic participants as well as increases the presence of *Bifidobacterium* spp in feces. Slight changes in the profile of fecal microbial metabolites were also reported. These data suggest that the cardioprotective effect of phenolic compounds could be mediated by the populations of bifidobacteria present in the gut microbiota [131]. The possible modulation of gut microbiota by olive oil and its role in cancer prevention, especially colorectal cancer, has also been suggested [148].

However, the complexity of the human diet, the lack of accuracy in the measurement of dietary intake and the extensive variation on microbiota between individuals challenges the evaluation of how diet changes modulate gut microbiota and its metabolic activity [149].

### 3.6. Other Bioactivities

The detailed description above was limited to the main bioactivities attributed to EVOO and its specific components. However, some other activities of biological relevance are under study. A recent extensive review highlighted the anti-aging properties of the major phenolic compound in EVOO, hydroxytyrosol, suggesting that it can contribute to the correct regulation of mechanisms that maintain cell homeostasis, such as mitogen-activated protein kinase (MAPK) and mammalian target of rapamycin (mTOR) pathways, whose imbalance is a hallmark of aging [150]. Moreover, this phenolic compound also modulates the metabolism of adipose tissue, stimulating mitochondrial biosynthesis and increasing the function of the mitochondrial respiratory chain in vitro [151].

EVOO consumption has also been associated with the enhancement of blood circulation and coagulation, by reducing platelet aggregation (mechanism related to CVD) and decreasing the levels of coagulation factor VII, effects attributed to minor components of EVOO [12]. Interestingly, a study carried out in murine models reported that polyphenols present in EVOO may improve learning and memory, by reversing the oxidative damage in the brain associated with aging and diseases related to the production of amyloid-β protein [152].

As a final remark, hydroxytyrosol, pinoresinol and oleuropein from EVOO have been reported to possess antimicrobial capacity. Pinoresinol has shown antifungal activity against several pathogenic fungi such as *Fusarium verticillioides*, *Fusarium graminearum* and *Candida albicans* [121]. Additionally, oleuropein and hydroxytyrosol were found to be effective against fungi and several strains of bacteria, viruses, including human immunodeficiency viruses (HIV) and parasites [151,153].

## 4. EVOO Quality Regarding the Chemical Composition

**Figure 4:**
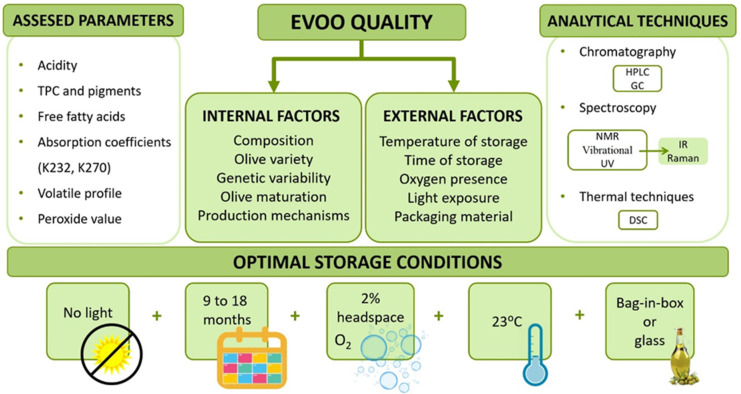
Parameters and factors concerning EVOO quality and its preservation.

As aforementioned, olive oil qualification as “extra virgin” demands various requisites. On one hand, it must be obtained using exclusively mechanical procedures, which grants the “virgin” label. On the other hand, it must show a low free acidity (<0.8%), low peroxide value, high antioxidant activity, a perceptible fruity taste and no sensory defects, acquiring in this way the label “extra”. However, once those badges are achieved, it is important to attend to some storage factors to maintain and assure these quality characteristics until the oil is used [154,155]. Figure 4 shows a summary concerning the quality-involved parameters.

### 4.1. Analytical Techniques

Parameters usually taken as EVOO quality indicators, as well as authenticity and fraud control markers are acidity, peroxide value, absorption coefficients K_232_ and K_270_, color, total phenolic content (TPC), fatty acids composition and volatile compounds’ profile. Spectrophotometric techniques are the most used to perform stability and quality analysis of EVOO since they are based on quick, simple, cheap and non-destructive methodologies offering the possibility of executing real-time controls. Several compounds are studied through these methodologies, such as certain pigments (chlorophylls, pheophytins, carotenoids, etc.) which are an easy and quick way to assess the degradation of EVOOs caused by temperature and/or light. The absorbance range analyzed for this purpose is between 350 and 750 nm [154,156].

Phenolic compounds, in addition of being responsible for a large part of the beneficial effects of EVOO, actively intervene in its quality, extending its useful life thanks to its antioxidant power [157]. This is the reason why its concentration is analyzed as a quality measure since the loss of these compounds is parallel to the degradation of EVOO. They are usually analyzed spectrophotometrically following the Folin–Ciocalteu method, as TPC (at absorbances of ~750 nm) and using gallic acid as standard, a methodology that offers speed, ease and reproducibility [155].

For the subsequent analysis of visible spectroscopy data and the prediction of optimal storage conditions, it is possible to use certain mathematical tools, such as multilayer perceptrons, capable of finding non-linear relationships between the variables involved, whether dependent or independent, based on artificial neural networks. Despite being a tool with many chemical applications including the food industry, it is important to add that it does not provide very reliable extrapolations since the model is based reliably on the breadth of the experimentally studied data [154,158].

Other analytical techniques that can be used are high-performance liquid chromatography or gas chromatography (HPLC, GC), nuclear magnetic resonance (NMR), differential scanning calorimetry (DSC) or vibrational spectrometry, which includes Raman and infrared spectrometry. These processes are more accurate when quantification is desired, although they are also more time-consuming, they require more specific types of equipment and are usually aimed at determining the origins of the EVOO or performing anti-fraud controls [159,160].

As an innovative and emerging methodology, it is worth highlighting the use of chemosensors and biosensors. These devices were initially based on electrochemical detection but have evolved in the direction of being coupled to smartphones, allowing to combine spectrophotometric techniques with optical image detection from the smartphone’s camera, resulting in on-site analysis of samples without requiring any pretreatment and without the need for a laboratory environment [155,161].

### 4.2. Effect of Olive Variety

As expected, the variety of olives used to produce EVOO determines its final composition, constituting an internal factor that influences stability and quality [162,163]. Not only the variety but also the geographical location and the growing conditions, as well as the applied mechanical production process, affect its quality [51,164,165]. Taking all these variables into account, it is possible to obtain EVOOs with different concentrations of compounds in such wide ranges as 50.5–80.5% for oleic acid, 91–665 mg/kg for α-tocopherol and 50–900 mg/kg for secoiridoid derivatives and lignans [166]. As an example, two varieties are discussed in this section, Empeltre varietal and Manzanilla Cacereña varietal. Based on the analyzes performed by visible spectrophotometry Manzanilla varietal contains a major amount of pigments (chlorophylls and their derivatives such as pheophytins, as well as carotenoids), and greater absorbance is observed on EVOO produced using Manzanilla Cacereña varietal. This fact also affects EVOO’s coloration, since the greater the number of pigments, the more yellowish-greenish the tonality. Likewise, a higher density is also observed in the liquid produced with the variety of olives that present a higher concentration of pigments [154].

Another study based on the comparison of EVOOs from two different varieties, Manzanilla and Picual, determined that the Manzanilla variety has a higher PUFA content and a lower MUFA content than the Picual variety, although a decrease in MUFA was found proportional to the increase in PUFA during degradation of both types of EVOO. Similarly, the Picual variety shows a higher content of polyphenols than Manzanilla, and therefore, a higher antioxidant activity. According to these authors, the compounds most influenced by the variety of olive used are fatty acids [167].

Therefore, the amount of oleic acid and natural antioxidant compounds, namely tocopherols and phenolic compounds and particularly, oleuropein, that are present in the final composition of the EVOO, is directly proportional to its better resistance to degradation. This fact is due to their capacity to avoid the formation of volatile compounds, responsible for the rancidness defect, and consequently enhance its shelf-life [166,168].

### 4.3. Effect of Light

Exposure to sunlight is one of the main factors that promote and accelerate the degradation of chemical compounds, such as pigments or phenolic compounds. Besides, it is one of the key factors as the EVOO is highly exposed to light during its stay in supermarkets and points of sale [169]. A study that analyzed the effect of light in 14 commercial varieties of EVOOs, recreating the conditions to which they are subjected in stores for 6 months, showed that light has a greater degrading effect on those compounds that contribute to the health-promoting properties, such as phenolic compounds, than on the compounds responsible for its quality, like acidity or oleic acid content. The quantification of the different analyzed compounds: oleic acid, total phenolic compounds, MUFA, PUFA, α-tocopherol, lignans and oleuropein, was carried out using HPLC and concluded that the loss of the “extra” condition can be predicted in time according to the initial concentration of oleuropein that a certain EVOO presents, thus this compound shows a strong antioxidative power [166]. Another fast and efficient way to perform light degradation analysis is by measuring pigments, such as chlorophyll. Nevertheless, light exposure is not recommended during the storage period if extending EVOO’s shelf-life and preserving its properties is desired [170,171].

### 4.4. Effect of Temperature

Another factor to consider during EVOO conservation is temperature. Several studies carried out with different EVOOs from different sources (Almazara del Ebro and As Pontis) highlight this fact. Comparing the absorption spectra in the visible ultraviolet of the oils, temperatures close to 40 °C cause greater adulterations in the composition of pigments than low temperatures, such as 3 °C, although the ideal temperature for its conservation is around 23 °C [154,172].

As a curious fact, when EVOO is stored at low temperatures, a crystallization process caused by freezing can be observed. In this process, triacylglycerols start to freeze through the methyl fraction, which is just the part that melts first in the opposite process. Through the analysis of the freezing and melting kinetics, information related to the geographical origin of the EVOO can be obtained [160,173]. Moreover, low temperatures application during EVOO processing has shown to be an effective strategy to improve its yield and quality [174].

### 4.5. Effect of Time

Time, as in most aspects of life, also plays a key role in the quality of the EVOO. Generally speaking, the shelf life of EVOOs is between 9 and 18 months, depending on other concomitant factors such as temperature or chemical composition, since when it is stored at 3 °C or room temperature, less degradation is observed than at 40 °C during the same period analyzed (88 days). In the same way, depending on the nature of the compounds that are present, they will suffer more or less degradation over time. Going back to previous examples, EVOOs obtained from the Empeltre and Manzanilla Cacereña varieties suffered different degradations under the same conditions: the Manzanilla Cacereña variety generated an EVOO whose visible absorption spectrum suffered a drastic decline in the first 10–12 days of storage after opening the bottle, regardless of temperature, while the Empeltre variety maintained a more linear spectrum over time, suggesting that the Manzanilla variety contained more easily degradable compounds [154]. Likewise, a longer study in time, in which the degradation suffered by 14 varieties of commercial EVOOs during 22 months of storage in the absence of light and at room temperature was analyzed, showed that peroxide values increase proportionally with storage time, so it is recommended that consumption occurs as close as possible to the production process [175].

### 4.6. Effect of Oxygen Presence

Oxygen presence is an essential factor in the oxidative degrading process. Moreover, EVOO oxygen content once produced, oxygen permeability while packaging and headspace oxygen are important factors influencing deterioration of lipids and overall EVOO quality and shelf-life during storage [169]. A study conducted on the effect of headspace oxygen concentration and the presence of light on the quality of EVOO over time determined that the shelf life of EVOO could be maximized, exceeding 12 months of stability, by applying headspace oxygen between 2% and 5%. However, intending to preserve the majority of beneficial compounds possible, such as pigments and polyphenols, it is preferable to carry out preservation in the absence of light and at low temperatures, around 10 °C, as well as using headspace oxygen of 2%. For this purpose, modified atmosphere packaging can be used, in which oxygen is replaced by other inert gases such as argon or helium [176].

### 4.7. Effect of Packaging Material

Finally, another external factor that influences the quality of the EVOO over time is the packaging material chosen. Some of the materials of choice for this purpose are tinted glass, polyethylene terephthalate (PET), tinplate, aluminum, tetra bricks or bag-in-box packages [177]. Various studies position glass as one of the materials that best preserves EVOO, since it is an inert material and, being tinted, reduces the passage of light. On the other hand, polypropylene and polyethylene are materials that show high oxygen permeability, so they are not recommended when preserving EVOO properties for a long time [169,178]. However, as happened before, this factor also depends on other conditions. The use of bag-in-box packages at home environment between 22 and 37 °C seems to benefit the preservation and lengthening of the EVOO shelf-life, as demonstrated in a study in which this packaging method was compared to tinplate steel containers [179].

## 5. Possible Applications of EVOO beyond Nutritional Purposes

### 5.1. Extractive Solvent

EVOO used in preparing culinary dishes offers numerous advantages, whether the implicit biological activities listed above or certain physicochemical characteristics that become indirectly beneficial. An example is the extractive capacity of some lipophilic compounds contained in the rest of the ingredients present in the recipes, managing to isolate them from the protection of the original matrix and, therefore, increase their bioavailability in the body, as occurs with lycopene present in tomato, the largest known source of this compound. This transfer of the lycopene from the tomato to the EVOO is achieved with a simple application of microwave heat to the food which additionally favors its isomerization, obtaining Z-Lycopene, the isomer that is mostly absorbed and is found in plasma. Hence, this EVOO capacity could be used to maximize extractive processes of certain lipophilic molecules, allowing their safe application in the food industry and avoiding the use of contaminating organic solvents [180].

### 5.2. Therapeutic Application

As previously mentioned, the bioactivities associated with EVOO are numerous, many related to its antioxidant capacity. The inclusion of EVOO in the daily diet supposes health benefits and helps to prevent the appearance of certain diseases such as heart and vascular, neurodegenerative, cytotoxic, metabolic and inflammatory diseases, among others, [3,181] due to its content in functional compounds such as polyphenols, tocopherols, carotenoids, sterols, fatty acids, squalene, etc. Therefore, EVOO becomes an attractive candidate for possible applications in therapeutics, constituting a potential strategy to use it in certain treatments that require its properties [182]. An example of this is a study conducted on the ability of EVOO to counteract the effect of chromium poisoning, a compound classified among the 20 most dangerous for humans and for the environment by the Agency for Toxic Substances and Disease Registry (ATSDR), which is capable of causing both acute and chronic toxicity, depending on the exposure. Some of the damages it causes in humans are neurotoxicity, genotoxicity, carcinogenicity, dermatotoxicity, or hepatotoxicity. So the oral administration of 300 µL of EVOO in animal models of rats previously intoxicated with chromium manages to reduce the genotoxic, immunotoxic and cytotoxic effects of the most harmful form of this compound, its hexavalent derivative, reducing the consequences of punctual or prolonged exposure [183].

Another way of approaching this point is the addition of more beneficial compounds to EVOO since it is a product daily consumed in many countries, which means transforming it into a vehicle enriched with functional compounds so that the health-promoting effects that EVOO already owns per se are increased [184]. An effective approach to incorporate such compounds into EVOO is using encapsulation, which preserves them from becoming rancid while improving the stability of those functional compounds such as vitamins or calcium [185,186,187]. 

## 6. Conclusions

EVOO, also colloquially known as “liquid gold” is a natural product of unquestionable value and not only in the monetary sense of the term but also for its recognized properties and advantages on health. It is considered food for its nutritional value and is practically a requirement of the MED, contributing to the benefits associated with it. Its chemical composition allows EVOO to be classified as a lipophilic product since lipids are the main compounds, especially MUFA, followed by PUFA. This lipid fraction is responsible for protective properties on coronary, autoimmune and inflammatory disorders, granting anti-thrombotic and regulation effects of blood pressure Although in a smaller quantity, other compounds such as tocopherols or polyphenols are also present, which are associated with the powerful antioxidant and inflammatory activity of EVOO, among other qualities. For all these reasons, the inclusion of this golden ingredient in the diet, in addition to offering characteristic organoleptic properties, provides substances capable of preventing the appearance or development of diseases of various nature, from heart and circulatory diseases to metabolic disorders, including carcinogenic processes. Compared to other types of olive oil, EVOO must meet more stringent requirements that give it the right to possess that nomenclature. However, it is a shame that, during distribution and storage, the previously achieved quality is corrupted, leaving some of its most distinguished and desirable properties on the way. Therefore, the factors that promote the degradation of its components must be known, intending to reduce the negative impact that may originate in EVOO and thus increasing its shelf-life. These factors can be divided into intrinsic factors, such as the variety of olives and their cultivation conditions, about which little can be done once EVOO has been produced; and external factors, which include exposure to light, temperature, time or the type of material used for packaging. Due to the functional properties it presents, other applications for EVOO could also be considered, such as its use in therapies in which other treatments are not very effective, like certain neurodegenerative diseases or as a vehicle for the administration of certain pharmacological compounds in a comfortable way, by simply adding them to the diet. Future studies aimed at optimizing and maximizing the capabilities and applications of this product will, without a doubt, be welcomed and well received by the industry, not only food and agriculture but also by the pharmaceutical and cosmetics industries.

## Figures and Tables

**Table 1:** Types of olive oils. Modified from EU Council Regulation (EC) No 1234/2007 [15].

Type of Oil		Characteristics	Free Acidity
**Virgin olive oils**	EVOO	They are characterized for being obtained by mechanical or other physical processes under specific thermal conditions that do not cause alterations in the oil and have not suffered any treatment other than washing, decantation, centrifugation or filtration. Excluded are oils obtained using solvents or adjuvants with chemical actions, by re-esterification process or any mixture with oils of other types.	<0.8 g per 100 g
	Virgin olive oil		≤2 g per 100 g
	Lampante olive oil		>2 g per 100 g
**Refined olive oil**		In this case, virgin olive oil is submitted to a refining process.	≤0.3 g per 100 g
***Olive oil*** **(composed of refined olive oils and virgin olive oils)**		It is the result of the blending of the two previous oils: virgin olive oils (not lampante oil) with refined olive oil.	≤1 g per 100 g
**Crude olive pomace oil**		This type refers to oil obtained from olive pomace by using solvents, physical treatments or oil corresponding to lampante olive oil type, except for certain specified characteristics. As well as in the case of virgin olive oils, excluded are oils obtained by means of re-esterification and mixtures with oils of other types.	
**Refined olive pomace oil**		This type is obtained from refining crude olive pomace oil.	≤0.3 g per 10 g
**Olive pomace oil**		It if the resultant oil from mixing refined olive pomace oil and virgin olive oil different than lampante oil.	≤1 g per 100 g

NOTES: (1) The International Olive Council (IOC) establishes olive oil standards in terms of sensory analysis and chemical composition for each category. (2) In the case of Lampante oil, it is also referred as “virgin olive oil not fit for consumption” by the IOC and with a free acidity higher than 3.3 g per 100 g.

**Table 2:** Major EVOO components.

Component		Concentration	References
**Lipids**			
Fatty acids (%)			
Myristic acid	C14:0	0.05	[53]
Palmitic acid	C16:0	9.4–19.5	[51,54]
Palmitoleic acid	C16:1	0.6–3.2	[51,54]
Heptadecanoic acid	C17:0	0.07–0.13	[51]
Heptadecenoic acid	C17:1	0.17–0.24	[51]
Stearic acid	C18:0	1.4–3.0	[51,54]
Oleic acid	C18:1	63.1–79.7	[51,54]
Linoleic acid	C18:2	6.6–14.8	[51,54]
α-Linolenic acid	C18:3	0.46–0.69	[51,54]
Arachidic acid	C20:0	0.3–0.4	[51,54]
Eicosenoic acid	C20:1	0.2–0.3	[51,54]
Docosanoic acid	C22:0	0.09–0.12	[51,54]
Lignoceric acid	C24:0	0.04–0.05	[51]
MUFA		65.2–80.8	[51]
PUFA		7.0-15.5	[51]
Other lipids			
Diacylglycerols (%)		1–2.8	[53]
Monoacylglycerols (%)		0.25	[53]
Total sterol content (mg/kg)		1000–3040	[43,55]
**Tocopherols (mg/kg)**			
α- Tocopherol		10.2–208	[51,54,56]
β- Tocopherol		0.75–1.05	[51]
γ- Tocopherol		0.7–2.1	[51]
**Carbohydrates (mg/kg)**			
Squalene		200–8260	[43,54,56,57]
**Pigments (mg/kg)**			
Total chlorophylls (mg/kg)		0.15–61.96	[51,58]
Pheophytin-a (mg/kg)		0.08–0.49	[56]
Total carotenoids (mg/kg)		0.53–31.51	[51,54,58]
β-carotene (mg/kg)		0.15–0.67	[56]
Lutein (mg/kg)		0.65–3.60	[56]
**Other Compounds**			
Total phenolic compounds (mg/kg)		213–450	[54]
Triterpene dialcohols (% of total sterols)		0.9–2.8	[55]
β-sitosterol (mg/kg)		530.2–2638.6	[56]

**Table 3:** Main bioactivities associated with EVOO consumption.

Bioactivity	Studies Description	Main Results	Ref
*Cardioprotection*	RCT, PREDIMED *(n* = 7447 participants at high CVD risk)	Following a MED enriched with EVOO decreases CVD risk by 30%	[30,107]
	PREDIMED observational study (*n* = 7216 participants)	For each 10g EVOO/day intake, CVD risk decreases by 10%	[112]
	Systematic review of 15 RCTs	10–50 mL/day EVOO reduced diastolic blood pressure by 0.7 mm Hg	[113]
	Meta-analysis of 9 studies (38,673 stroke and 101,460 CHD cases from RCT, case-control and prospective studies)	For every increase of 25 g of olive oil consumption the risk of CVD, stroke and CHD was reduced by 18%, 26% and 4% respectively	[114]
	Meta-analysis of 26 RCTs	High polyphenol olive oil intake significantly reduced CVD and inflammatory markers	[115]
*Antioxidant properties*	European Food Safety Authority health claim.	5 mg/day of olive oils polyphenols protects blood lipids from oxidation	[116]
	RCTs evaluating the effect of olive oils consumption on blood lipids oxidative state.	EVOO and high-phenolic olive oils consumption reduces LDL oxidation in a dose-dependent manner	[117,118,119,120]
	Controlled trials with sub-samples of PREDIMED cohort (*n* = 296) and (*n* = 210)	Adherence to a MED enriched with EVOO improves HDL function and protects against LDL oxidation	[108,109]
	In vitro studies review.	Lignans present in EVOO show antioxidant activity in vitro	[121]
*Anti-inflammatory capacity*	Meta-analysis of 13 studies based on 9 RCTs	Regular consumption of EVOO reduces IL-6, CRP and TNF-α levels	[122]
	Meta-analysis of RCTs (3106 participants)	Diet supplemented or enriched in olive oil reduces IL-6 and CRP plasmatic levels	[123]
	Randomized crossover study (49 patients)	High-phenolic virgin olive oil in breakfast reduces postprandial inflammatory response.	[124]
*Antitumoral activity*	Meta-analysis of 19 case-control studies (comprising 13,800 cancer cases and 23,340 controls)	Olive oil consumption is associated with lower odds of developing digestive and breast cancers	[125]
	Cohort-study follow up (2321 breast cancer cases and 1665 controls) and meta-analysis	Inverse association between adherence to MED and ERN breast cancer	[126]
	RCT with a sub-sample of the PREDIMED cohort (*n* = 4152 women)	Women following MED enriched in EVOO showed 62% relatively lower risk of breast cancer compared to control low-fat diet	[110]
	Systematic review and meta-analysis of 83 studies, comprising a total of 2,130,753 subjects	The adherence to MED is associated with lower risk of cancer mortality and lower risk of breast, colorectal, gastric and liver cancers, among others	[127]
	In vitro experiments of antitumoral activity of phenolic compounds on cancer cell lines	The phenolic fraction of EVOO, as well as isolated phenolic compounds, shows antitumoral and cytotoxic effect on different cancer cell lines	[128,129,130]
*Gut microbiota modulation*	RCT with 12 hypercholesterolemic participants	Virgin olive oil enriched in phenolic compounds consumption favors gut bifidobacteria growth and decreases serum levels of oxidized LDL	[131]
	Systematic review and meta-analysis of 17 RCTs	Polyphenols exert a prebiotic action on gut microbiota, improving also CVD and CRC	[132]

EVOO: extra virgin olive oil; CVD: cardiovascular disease; CHD: coronary heart disease; RCT: randomized controlled trial; MED: Mediterranean diet; HDL: high-density lipoprotein; LDL: low-density lipoprotein; IL-6: interleukin-6; CRP: C-reactive protein; TNF-α: tumor necrosis factor alpha; IBD: inflammatory bowel disease; ERN: estrogen receptor negative; CRC: colorectal cancer.

## Abbreviations List

**Generic**	
CHD	Coronary Heart Disease
CVD	Cardiovascular Diseases
DSC	Differential Scanning Calorimetry
DPPH	2,2-Diphenyl-1-picryl-hydrazyl-hydrate free radical assay
EFSA	European Food Safety Authority
EU	European Union
EVOO	Extra Virgin Olive Oil
GC	Gas Chromatography
HPLC	High Performance Liquid Chromatography
HIV	Human Immunodeficiency Viruses
IC_50_	Half Maximal Inhibitory Concentration
IOC	International Olive Council
MED	Mediterranean Diet
NMR	Nuclear Magnetic Resonance
PREDIMED	Prevention Through Mediterranean Diet
USA	United States of America
VOO	Virgin Olive Oil
**Biological and Chemical Compounds**	
CRP	C-Reactive Protein
DNA	Deoxyribonucleic Acid
HDL	High-Density Lipoprotein
Hg	Mercury
IL-6	Interleukin-6
LDL	Low-Density Lipoprotein
MAPK	Mitogen-Activated Protein Kinase
miRNA	Micro Ribonucleic Acid
mTOR	Mammalian Target of Rapamycin
MUFA	Monounsaturated Fatty Acids
OOL	Oleic–Oleic–Linoleic Triacylglycerol
OOO	Oleic–Oleic–Oleic Triacylglycerol
PET	Polyethylene Terephthalate
POO	Palmitic-Oleic-Oleic Triacylglycerol
POL	Palmitic–Oleic–Linoleic Triacylglycerol
PUFA	Polyunsaturated Fatty Acids
SOO	Stearic–Oleic–Oleic Triacylglycerol
TPC	Total Phenolic Content
TNF-α	Tumor Necrosis Factor-A
